# Outcome of Transfer Time Difference From Diagnosis to Operation Room in Acute Type A Aortic Dissection Complicated by Malperfusion

**DOI:** 10.1016/j.atssr.2025.05.015

**Published:** 2025-06-09

**Authors:** Chikashi Nakai, Andrew Ku, Yuan Haw Wu, Junyi Liu, Nikhil Azhagiri, Eduardo Danduch, Saeed Tarabichi, Li Zhang, Sanjay Samy

**Affiliations:** 1 Department of Cardiothoracic Surgery, Albany Medical Center, New York, New York

## Abstract

**Background:**

There are few reports of time effect for postoperative outcomes in patients with acute type A aortic dissection (ATAAD) complicated by malperfusion syndrome (MPS), especially transfer time difference from diagnosis to operation room (OR). To elucidate whether time between diagnosis and OR might be a significant factor, this study evaluated surgical outcomes of ATAAD complicated by MPS.

**Methods:**

Between October 2013 and June 2024, 159 patients with ATAAD underwent emergent aortic repair; 54.7% (87/159) presented with MPS, 45.3% (72/159) without MPS. Of 87 patients with MPS, 69.0% (60/87) were transferred to the OR within 150 minutes from initial diagnosis (immediate repair), whereas 31.0% (27/87) were transferred to the OR after 150 minutes (late repair).

**Results:**

In the MPS group, there was a significant difference in 30-day mortality rate between immediate and late repair, 20.0% (12/60) vs 48.1% (13/27; *P* < .01). Mean follow-up time was 33.0 ± 35.8 months. Cumulative survival rate in 5 years of patients with MPS was 64.6% for immediate repair and 46.1% for late repair. A significant difference was noted in long-term outcome between the groups (*P* = .03), whereas there was no difference in the non-MPS group (*P* = .11). On multivariable Cox regression analysis, age >65 years, cardiac tamponade, and late aortic repair were associated with increased long-term mortality (*P* = .02, .02, and <.01).

**Conclusions:**

Immediate transfer from diagnosis to OR significantly improved long-term outcome in patients with ATAAD complicated by MPS. Older age and preoperative cardiac tamponade worsened long-term mortality in this cohort.

**In Short**

▪ Immediate transfer from diagnosis to operation room significantly improved long-term outcomes in patients with acute type A aortic dissection complicated by malperfusion syndrome.
▪ Older age, preoperative cardiac tamponade, and delayed transfer were predictors of increased long-term mortality.

Time interval between onset or diagnosis of acute type A aortic dissection (ATAAD) and emergent aortic repair has been a significant factor in postoperative outcomes.^1^^,^^2^ When patients were transferred to the operation room (OR) for emergent aortic repair within 240 minutes of diagnosis, short-term outcome was better than that of late repair.^1^ Immediate aortic repair within 5 hours of symptom onset improved long-term outcomes in patients with preoperative malperfusion syndrome (MPS).^2^ However, there are few reports about time effect for postoperative outcomes in patients with MPS, especially transfer time from diagnosis to OR. We focused on postoperative outcomes of transfer time difference from diagnosis to OR in patients with ATAAD complicated by MPS.

## Patients and Methods

**Figure 1:**
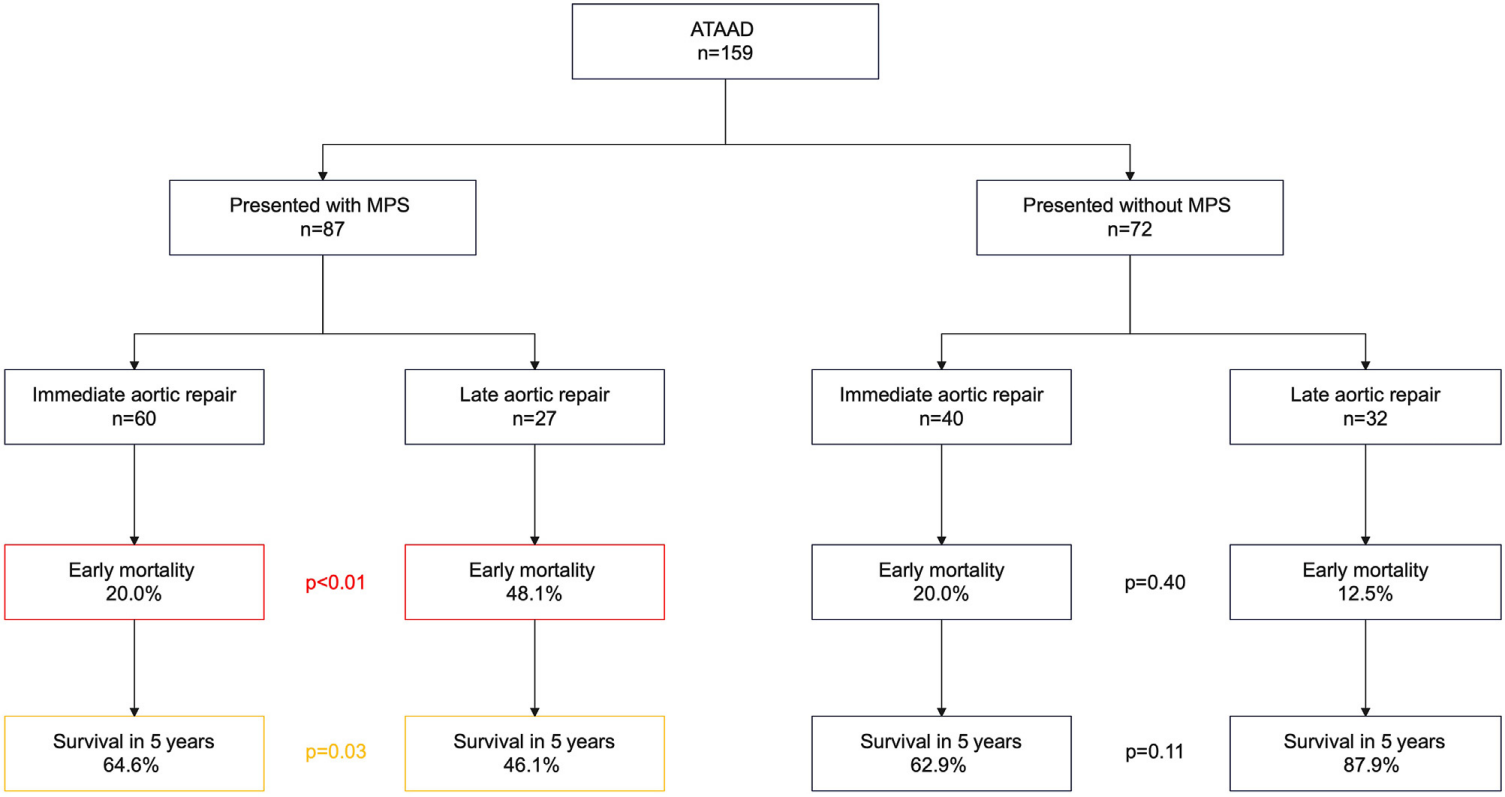
The 159 patients with acute type A aortic dissection (ATAAD) were divided into 2 groups, malperfusion syndrome (MPS) and non-MPS. Of 87 patients with MPS, 60 underwent immediate aortic repair, whereas 27 had late aortic repair.

From October 2013 to June 2024, 159 patients with ATAAD underwent emergent aortic repair at Albany Medical Center, including 87 patients (54.7%) complicated by MPS. Of 87 patients with MPS, 60 were transferred to the OR within 150 minutes from diagnosis of ATAAD (immediate repair), whereas 27 were transferred to the OR after 150 minutes from diagnosis (late repair; Figure 1). Diagnosis of ATAAD was confirmed with computed tomography with contrast enhancement or transesophageal echocardiography. MPS is defined in Supplemental Table 1. In our strategy for MPS, central aortic repair was performed in all patients with ATAAD. Only 1 patient was transferred to the catheterization room first with acute coronary syndrome and diagnosed with ATAAD. We analyzed postoperative outcomes in patients with MPS compared by immediate vs late repair. The primary end point was 30-day mortality and cumulative survival rate in 5 years.

### Operative Procedure

The aortic repairs were conducted through a median sternotomy with full heparinization and cardiopulmonary bypass. The femoral artery, right axillary artery, or ascending aorta was accessed for arterial cannulation. Once the patient was cooled down for 45 minutes and reached a bladder temperature of 22 °C, cardiopulmonary bypass was discontinued, and circulation was arrested with or without antegrade cerebral perfusion. The decision of whether to perform antegrade cerebral perfusion was made by each surgeon. The intimal tear was resected, and hemiarch replacement was performed. After reconstruction of the distal ascending aorta or distal arch, cardiopulmonary bypass flow was resumed with systemic warming until the body temperature was 36 °C.

### Statistical Analysis

Categorical variables were described as numbers and percentages; continuous variables were presented as mean ± SD. Student *t*-test was performed for continuous variables that have a normal distribution and Mann-Whitney *U* test for those that do not have a normal distribution. Categorical variables were assessed by *χ*^2^ test and Fisher exact test. A value of *P* < .05 was considered statistically significant. Five predictors (age, brain malperfusion, coronary malperfusion, cardiac tamponade, and immediate aortic repair) for 30-day and long-term mortality were determined according to previous reports. Logistic regression analysis was used to evaluate the predictors of early mortality. The model fitting was confirmed by area under the receiver operating characteristic curve. The Kaplan-Meier method was carried out to evaluate the cumulative survival, and statistical differences were analyzed by log-rank test. Cox proportional hazards analysis was conducted to evaluate the predictors of long-term mortality. The proportional hazards assumption was verified by stratified log minus log plots. Data analyses were performed by SPSS version 29.0.0.0 software (IBM) and GraphPad Prism version 10.4.1 (GraphPad Software Inc).

## Results

Characteristics of patients with preoperative MPS are listed in Table 1; intraoperative and postoperative data are listed in Table 2. Preoperative characteristics in the MPS group were similar between the immediate and late repairs. The rate of brain malperfusion was significantly higher in the immediate repair group (*P* = .03), whereas coronary malperfusion, renal malperfusion, mesenteric ischemia, and cardiac tamponade did not differ between the groups. The mean transfer time from diagnosis to OR was 104.9 ± 41.0 minutes in the immediate repair group and 400.8 ± 316.2 minutes in the late repair group. Intraprocedural data were similar between the groups.

**Table 1:** Preoperative Characteristics of Patients With Malperfusion Syndrome

Characteristic	Immediate Repair (n = 60)	Late Repair (n = 27)	*P*
Age, y	62.2 ± 13.5	61.0 ± 16.7	.72
Age >65 years	26 (43.3)	11 (40.7)	.82
Male	45 (75.0)	17 (63.0)	.25
Brain malperfusion	14 (23.3)	1 (3.7)	.03
Coronary malperfusion	5 (8.3)	1 (3.7)	.66
Renal malperfusion	13 (21.7)	9 (33.3)	.25
Mesenteric ischemia	3 (5.0)	5 (18.5)	.10
Peripheral malperfusion	19 (31.7)	8 (29.6)	.85
Cardiac tamponade	25 (41.7)	13 (48.1)	.57
Time from diagnosis to operation room, min	104.9 ± 41.0	400.8 ± 316.2	<.01

Categorical variables are presented as number (percentage). Continuous variables are presented as mean ± SD.

**Table 2:** Procedural Characteristics and Postoperative Outcomes of Patients With Malperfusion Syndrome

Variable	Immediate Repair	Late Repair	*P*
Procedural characteristic			
Hemiarch replacement	56 (93.3)	21 (77.8)	.06
Total arch replacement	0 (0)	0 (0)	…
Aortic root replacement	4 (6.7)	5 (18.5)	.13
Coronary artery bypass grafting	8 (13.3)	4 (14.8)	.55
Cardiopulmonary bypass time, min	211.7 ± 75.9	225.1 ± 102.1	.50
Aortic cross-clamp time, min	130.3 ± 48.6	128.1 ± 62.2	.87
Circulatory arrest time, min	34.4 ± 16.3	31.0 ± 18.2	.42
Antegrade cerebral perfusion	29 (48.3)	15 (55.6)	.53
Postoperative outcome			
Stroke	12 (20.0)	6 (22.2)	.81
New dialysis requirement	6 (10.0)	6 (22.2)	.13
Length of hospital stay, d	15.4 ± 14.1	11.7 ± 10.8	.24
30-day mortality	12 (20.0)	13 (48.1)	<.01
Follow-up, mo	34.6 ± 32.8	21.6 ± 34.2	.10
Cumulative survival in 5 years, %	64.6	46.1	.03

Categorical variables are presented as number (percentage). Continuous variables are presented as mean ± SD.

### Early Surgical Outcomes

In patients with MPS, postoperative complications and length of hospital stay did not differ between the groups. The 30-day mortality rate was 20.0% in the immediate repair group and 48.1% in the late repair group. There was a significant difference in the 30-day mortality between the groups (*P* < .01). The cause of hospital death in each group is summarized in Supplemental Table 2. In the immediate repair group, 12 patients died within 30 days of multiple organ failure in 7 patients, stroke in 3 patients, and exsanguination in 2 patients. In the late repair group, 13 patients died within 30 days of undergoing aortic repair. The cause of death included multiple organ failure in 7 patients, heart failure in 1 patient, stroke in 2 patients, sepsis in 1 patient, mesenteric ischemia in 1 patient, and exsanguination in 1 patient. Multiple organ failure was related to extent of dissection, including acute kidney injury, liver failure, respiratory failure, and severe refractory acidosis (Supplemental Table 2). Exsanguination was caused by coagulopathy and significant bleeding from anastomosis sites. On logistic regression analysis, immediate aortic repair significantly improved early mortality (*P* < .01), whereas coronary malperfusion and cardiac tamponade were significant predictors of early mortality (*P* = .02 and *P* = .01; Supplemental Table 3).

### Long-Term Outcomes

**Figure 2:**
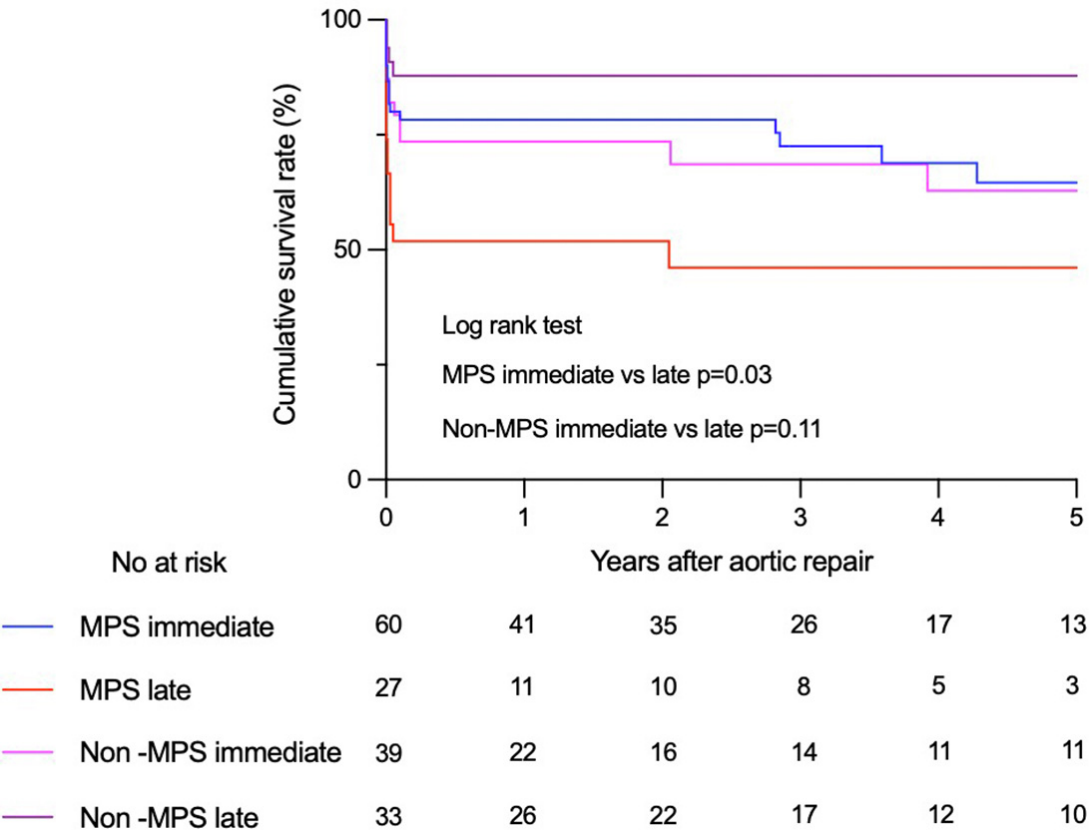
Cumulative survival rates of patients with malperfusion syndrome (MPS) and without MPS. In the patients with MPS, there was a significant difference in the long-term survival between the immediate and late repair groups (*P* = .03). In patients without MPS, there was no significant difference in the long-term survival between the immediate and late repair groups (*P* = .11).

The mean follow-up period was 33.0 ± 35.8 months. During the period, 8 patients with MPS died of heart failure in 2 patients, subdural hematoma in 1 patient, and unknown cause in 5 patients. The cumulative survival rate in 5 years in the immediate and late repair groups was 64.6% and 46.1%, respectively. A significant difference was noted between the groups (*P* = .03; Figure 2). The Cox proportional hazards analysis demonstrated immediate aortic repair to be a significant predictor of long-term survival (*P* < .01), although age >65 years and cardiac tamponade raised the long-term mortality (*P* = .02; Supplemental Table 3).

### Non-MPS group

Characteristics and intraoperative and postoperative data of patients without preoperative MPS are listed in Supplemental Table 4. The 30-day mortality and cumulative survival rate were not different between the immediate and late repair groups (*P* = .53 and *P* = .11; Supplemental Table 4; Figure 2).

## Comment

These are findings that can be summarized from our study.

• Early and long-term mortality in patients with MPS was significantly lower in the immediate repair group.
• Coronary malperfusion, cardiac tamponade, and late aortic repair were predictors of early mortality.
• Immediate aortic repair was a significant predictor for improved long-term survival, whereas older age and cardiac tamponade worsened long-term outcomes.
• Patients without MPS showed no significant difference in short- and long-term outcomes between the immediate and late repairs.

Direct admission from the helipad or emergency department to the OR allows a reduction in the interval between diagnosis and treatment.^3^ Between emergency department arrival and the start of aortic repair, 47.3% of patients with ATAAD experienced delays.^4^ Median time between arrival at the cardiac center and the start of aortic repair was 2.4 hours.^4^ However, little evidence is available about transfer time in patients with ATAAD complicated by MPS. Further studies are required to clarify how transfer time, including time from onset to diagnosis and diagnosis to the OR, affects postoperative outcomes.

The 30-day mortality rate in patients with MPS has been reported to range from 20.0% to 35.0%.^2^^,^^5^ Those results are in agreement with 28.7% in our study, although the 30-day mortality of the immediate repair group was lower than that of the late repair group (20.0% vs 48.1%). Most patients with MPS died of multiple organ failure in our study. It is our firm belief that immediate aortic repair decreased ischemia time of each organ and shock time secondary to cardiac tamponade and coronary malperfusion. Immediate aortic repair within 5 hours from onset to the OR revealed improved long-term outcomes with 76.7% cumulative 5-year survival.^2^ The cumulative 5-year survival in our study was 64.6%, which was consistent with the previous study.

In patients who were hemodynamically stable with mesenteric MPS, an endovascular approach and delayed open repair revealed favorable outcomes.^6^ When transfer is delayed, an endovascular approach for stable patients might be one option to improve mortality. However, direct transfer to a hybrid OR for proximal aortic repair first remains the “gold standard” for patients with MPS, given the risk of aortic rupture, and an endovascular approach for distal malperfusion should be considered at the same time or subsequently.^7^ Any patients who are transferred late may benefit from transfer to a hybrid OR to prepare for potential aortic repair and endovascular approach.

Patients who are transferred to the OR late without MPS demonstrated the highest short- and long-term survivals in our study. This could be explained by the fact that stable patients without MPS or shock might survive regardless of transfer time.

This study has several limitations. First, it is a retrospective single-center study. A multicenter study is needed in the future because the transfer system and surgical management might differ in each facility. Second, some patients were lost during the follow-up periods. As a result, the number of remaining patients decreased, which might cause lower power statistically. Third, most patients were transferred to Albany Medical Center from an outside hospital, and some data were missing with regard to the onset time of ATAAD, although all of transfer time from diagnosis to OR was confirmed.

### Conclusion

Immediate transfer from diagnosis to OR significantly improved long-term outcomes in patients with ATAAD complicated by MPS. Older age, preoperative cardiac tamponade, and delayed transfer were predictors of increased long-term mortality in this cohort.
