# Supplementary material for: Outcome of Transfer Time Difference From Diagnosis to Operation Room in Acute Type A Aortic Dissection Complicated by Malperfusion
Source: Ann Thorac Surg Short Rep. 2025 Jun 9;3(4):974–8. doi: 10.1016/j.atssr.2025.05.015 (PMC12712158; doi:10.1016/j.atssr.2025.05.015)
Supplement: Supplementary Table 1 [file mmc1.docx]

**Supplemental Table 1 Definition of MPS**

| Malperfusion | Deifinition |
| --- | --- |
| Brain malperfusion | Disturbed consciousness or paralysis in patients with common carotid artery dissection on preoperative CT |
| Coronary malperfusion | ST-elevation on 12-lead electrocardiogram with hypokinesis at the corresponding echocardiogram region with dissection in the right coronary artery, left coronary artery, or both on preoperative CT |
| Renal malperfusion | Preoperative creatinine > 1.0 mg/dL with poor kidney contrast on preoperative CT |
| Mesenteric ischemia | Abdominal pain with severe stenosis or occlusion of the celiac artery, superior mesenteric artery, or both on preoperative CT |
| Peripheral malperfusion | Ischemic pain of upper or lower limb with disappearance of blood flow to the brachial or femoral artery |

CT: computed tomography, MPS: malperfusion syndrome
