# Supplementary material for: Outcome of Transfer Time Difference From Diagnosis to Operation Room in Acute Type A Aortic Dissection Complicated by Malperfusion
Source: Ann Thorac Surg Short Rep. 2025 Jun 9;3(4):974–8. doi: 10.1016/j.atssr.2025.05.015 (PMC12712158; doi:10.1016/j.atssr.2025.05.015)
Supplement: Supplementary Table 2 [file mmc2.docx]

**Supplemental Table 2 Cause of hospital death in patients with ATAAD**

| Cause of death | MPS immediate  n=12 | MPS late  n=13 | Non-MPS immediate  n=8 | Non-MPS late  n=4 |
| --- | --- | --- | --- | --- |
| Multiple organ failure, n(%) | 7 (58.3) | 7 (53.8) | 4 (50.0) | 2 (50.0) |
| Stroke, n(%) | 3 (25.0) | 2 (15.4) | 1 (12.5) | 1 (25.0) |
| Exsanguination, n(%) | 2 (16.7) | 1 (7.7) | 1 (12.5) | 0 (0) |
| Heart failure, n(%) | 0 (0) | 1 (7.7) | 1 (12.5) | 0 (0) |
| Sepsis, n(%) | 0 (0) | 1 (7.7) | 1 (12.5) | 1 (25.0) |
| Mesenteric ischemia, n(%) | 0 (0) | 1 (7.7) | 0 (0) | 0 (0) |

ATAAD: acute type A aortic dissection, MPS: malperfusion syndrome
