# Supplementary material for: Outcome of Transfer Time Difference From Diagnosis to Operation Room in Acute Type A Aortic Dissection Complicated by Malperfusion
Source: Ann Thorac Surg Short Rep. 2025 Jun 9;3(4):974–8. doi: 10.1016/j.atssr.2025.05.015 (PMC12712158; doi:10.1016/j.atssr.2025.05.015)
Supplement: Supplementary Table 3 [file mmc3.docx]

**Supplemental Table 3 Multivariable analysis for early and long-term mortality in patients with ATAAD**

| Risk factors | Adjusted Odds Ratio | 95% Confidence Interval | *p* |
| --- | --- | --- | --- |
| Logistic regression |  |  |  |
| Age>65 years-old | 2.15 | 0.71 – 6.56 | 0.18 |
| Brain malperfusion | 2.59 | 0.53 – 12.59 | 0.24 |
| Coronary malperfusion | 12.16 | 1.46 – 101.70 | 0.02 |
| Cardiac tamponade | 4.84 | 1.40 – 16.79 | 0.01 |
| Immediate aortic repair | 0.16 | 0.05 – 0.54 | <0.01 |
| Risk factors | Adjusted Hazard Ratio | 95% Confidence Interval | *p* |
| Cox hazard proportional |  |  |  |
| Age>65 years-old | 2.57 | 1.20 – 5.54 | 0.02 |
| Brain malperfusion | 1.93 | 0.74 – 5.00 | 0.18 |
| Coronary malperfuison | 3.88 | 0.96 – 15.66 | 0.06 |
| Cardiac tamponade | 2.53 | 1.14 – 5.60 | 0.02 |
| Immediate aortic repair | 0.30 | 0.13 – 0.67 | <0.01 |

ATAAD: acute type A aortic dissection
