# Supplementary material for: Outcome of Transfer Time Difference From Diagnosis to Operation Room in Acute Type A Aortic Dissection Complicated by Malperfusion
Source: Ann Thorac Surg Short Rep. 2025 Jun 9;3(4):974–8. doi: 10.1016/j.atssr.2025.05.015 (PMC12712158; doi:10.1016/j.atssr.2025.05.015)
Supplement: Supplementary Table 4 [file mmc4.docx]

**Supplemental Table 4 Preoperative, procedural characteristics and postoperative outcoems in patients without MPS**

| Characteristic | Immediate repair n=39 | Late repair n=33 | *p* |
| --- | --- | --- | --- |
| Age, y | 59.8±13.9 | 59.8±13.8 | 0.99 |
| Male, n(%) | 23 (59.0) | 23 (69.7) | 0.35 |
| Time from dianogsis to operation room, minutes | 94.2±39.2 | 419.2±371.4 | <0.01 |
| Operative procedures |  |  |  |
| Hemiarch replacement, n(%) | 30 (76.9) | 23 (69.7) | 0.49 |
| Total arch replacement, n(%) | 0 (0) | 2 (6.1) | 0.21 |
| Antegrade cerebral perfusion, n(%) | 23 (59.0) | 18 (54.5) | 0.71 |
| Postoperative outcomes |  |  |  |
| Stroke, n(%) | 5 (12.8) | 2 (6.1) | 0.44 |
| Length of hospital stay, days | 12.3±11.0 | 13.1±13.2 | 0.80 |
| 30day mortality, n(%) | 8 (20.5) | 4 (12.1) | 0.53 |
| Follow-up term, months | 31.7±38.2 | 41.3±37.0 | 0.29 |
| Cumulative survival in 5years, % | 62.9 | 87.9 | 0.11 |

MPS: malperfusion syndrome
